# Additional Resistant Starch from One Potato Side Dish per Day Alters the Gut Microbiota but Not Fecal Short-Chain Fatty Acid Concentrations

**DOI:** 10.3390/nu14030721

**Published:** 2022-02-08

**Authors:** Peter DeMartino, Emily A. Johnston, Kristina S. Petersen, Penny M. Kris-Etherton, Darrell W. Cockburn

**Affiliations:** 1 Department of Food Science, Pennsylvania State University, University Park, PA 16802, USA; pzd115@psu.edu; 2 Department of Nutritional Sciences, Pennsylvania State University, University Park, PA 16802, USA; ejohnston@chsu.edu (E.A.J.); kristina.petersen@ttu.edu (K.S.P.); pmk3@psu.edu (P.M.K.-E.); 3 Department of Nutritional Sciences, Texas Tech University, Lubbock, TX 79409, USA

**Keywords:** resistant starch, gut microbiota, butyrate, potatoes

## Abstract

The composition of the gut microbiota and their metabolites are associated with cardiometabolic health and disease risk. Intake of dietary fibers, including resistant starch (RS), has been shown to favorably affect the health of the gut microbiome. The aim of this research was to measure changes in the gut microbiota and fecal short-chain fatty acids as part of a randomized, crossover supplemental feeding study. Fifty participants (68% female, aged 40 ± 13 years, BMI 24.5 ± 3.6 kg/m^2^) completed this study. Potato dishes (POT) contained more RS than refined grain dishes (REF) (POT: 1.31% wet basis (95% CI: 0.94, 1.71); REF: 0.73% wet basis (95% CI: 0.34, 1.14); *p* = 0.03). Overall, potato dish consumption decreased alpha diversity, but beta diversity was not impacted. Potato dish consumption was found to increase the abundance of *Hungatella xylanolytica*, as well as that of the butyrate producing *Roseburia faecis*, though fecal butyrate levels were unchanged. Intake of one potato-based side dish per day resulted in modest changes in gut microbiota composition and diversity, compared to isocaloric intake of refined grains in healthy adults. Studies examining foods naturally higher in RS are needed to understand microbiota changes in response to dietary intake of RS and associated health effects.

## 1. Introduction

The gut microbiome has been implicated in the etiology of preventable chronic disease, such as obesity and type 2 diabetes [1,2]. Gut health is affected by several factors, including genetics, the environment, and diet [3]. Overall diet quality and adherence to the Dietary Guidelines for Americans is poor in the US across age groups [4]. Dietary fiber intake is important for disease prevention [5] and for the health of the gut microbiome [6]; average fiber intake in the US is 16.4 g per day, well below the adequate intake of 14 g/1000 kcal/d [7]. Greater understanding of alterations to the gut microbiota and their metabolites induced by dietary changes will further disease prevention and management. 

When indigestible carbohydrates are fermented by the gut microbiota, short-chain fatty acids (SCFA) are produced. SCFA are both energy sources for the gut bacteria and host, as well as signaling molecules that play a role in energy metabolism [8]. The most abundant SCFAs are butyrate, acetate, and propionate, and these are associated with reductions in risk of cardiometabolic disease, some bowel disorders, and certain cancers [9,10,11,12]. Increasing intake of fermentable carbohydrates, is a key strategy to increase SCFA production and improve human health.

Resistant starch (RS) is starch that, due to structural differences from digestible starch, cannot be degraded by enzymes in the human gastrointestinal tract but can be fermented by colonic bacteria [13]. This starch exhibits a lower glycemic index [14] and is a substrate for gut microbiota [13], giving it potential for supporting human health. There are four main types of RS based on the factors that make it resistant to digestion. Potato RS is typically found as either the RS2 (intact starch granules in raw or incompletely gelatinized cooked potatoes) or RS3 (retrograded starch) in cooked and cooled products such as potato salad [15]. In each of these cases, the starch exhibits crystallinity that reduces digestibility by human enzymes [16].

RS supplementation significantly increases fecal butyrate concentration in humans [17,18], mice [19], and during in vitro fermentation with fecal inocula [20]. Intake of RS in the US is estimated to be less than 6 g/day [21]. However, most studies assess levels much higher than this (>20 g/day), typically relying on RS supplementation in the form of raw starch [17,18,22]. Even in studies where foods are prepared with RS, the amount of RS per item is greater than typical daily intake [23,24]. These doses of supplemental RS induce changes to the gut microbiota and SCFA levels, although these results are not generalizable to the general free-living population. Investigation of the effect of lower levels of RS intake is needed to determine the lower threshold for inducing favorable changes to the gut microbiota. 

The aim of this study was to determine the effect on the gut microbiota and fecal SCFAs of daily intake of a side dish containing one potato (averaging 145 g), compared to an isocaloric amount of refined grains in healthy adults. Changes in the diversity and composition of the microbiota were evaluated. It was hypothesized that a small increase in RS intake, from cooked potatoes, would increase the abundance of RS degrading and butyrate-producing bacteria, thereby significantly increasing fecal SCFA concentrations, compared to isocaloric intake of refined grains. 

## 2. Materials and Methods

### 2.1. Study Design

**Figure 1:**
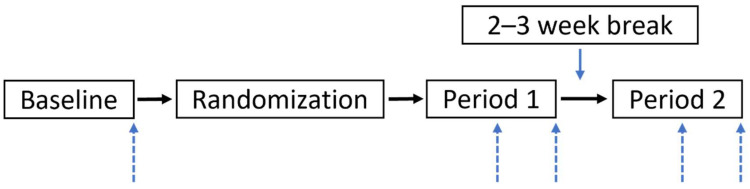
Study design. A randomized cross-over study was conducted where participants consumed a refined grain side dish and a non-fried potato side dish in random order daily for 4 weeks each, with a 2–3-week washout period in between conditions. Up arrows signify fecal sample collection days at baseline and at the midpoint and end of each condition (weeks 2 and 4).

The details of the trial design, conditions, and cardiometabolic and diet-related endpoints are reported elsewhere [25]. Here, we report results of analyses examining microbial diversity and composition and fecal SCFA, pre-specified secondary outcomes. Briefly, this was a single-blind, randomized, crossover trial, comparing the impact of intake of a potato-based side dish with a daily refined grain side dish in healthy adults. Participants were provided with one potato-based side dish (POT), or one control refined grain-based side dish (REF) daily, for four weeks, separated by a minimum two-week break (Figure 1). Dishes were calorie- and carbohydrate-matched and prepared without excess saturated fat or sodium. Participant compliance, assessed on a weekly basis using checklists, was high, with subjects reporting consumption of study dishes on 98% of study days, on average. All procedures involving human participants were approved by the Penn State Institutional Review Board (STUDY00007854). Participants provided written informed consent at screening prior to enrollment. This trial is registered at ClinicalTrials.gov (accessed on 15 December 2021) (identifier: NCT03495284).

### 2.2. Participants

Fifty adults were recruited from the State College, PA area through distribution of flyers at local businesses, listservs, and through the website of the Penn State’s Cardiometabolic Nutrition lab. Participants were healthy non-smokers between the ages of 25 and 75 years with a body mass index (BMI) between 20 and 40 kg/m^2^. Volunteers were excluded if they had a history of chronic disease, inflammatory conditions, GI disorders, or were taking any medications for these or if they reported an allergy or intolerance to any of the study dish ingredients. Detailed study inclusion and exclusion criteria have been published [25].

### 2.3. Study Dishes

Once weekly, participants reported to the Metabolic Diet Study Center on the Penn State Campus to pick up a cooler with their study dishes (six frozen dishes and one refrigerated dish per week). Dishes were prepared to limit saturated fat, sugar, and sodium, containing minimal amounts of other ingredients (i.e., scallions, spinach, garlic), and were calorie- and carbohydrate-matched. The dishes in both conditions contained 200 calories on average, with similar macronutrient (fat (3.7 g POT, 4.1 g REF), saturated fat (0.5 g POT, 0.7 g REF), carbohydrates (37 g POT, 34 g REF), protein (6.2 g POT, 6.0 g REF)) and sodium (228 mg POT, 236 mg REF) content. The dishes differed in fiber (3.6 g POT, 1.5 g REF) and potassium (826 mg, POT, 119 mg REF) content. Nutrient values were determined using Food Processor^®^ (ESHA, Salem, OR, USA). Participants were instructed to consume only potatoes provided for the study during the potato condition and to avoid all potatoes during the refined grain condition; they incorporated side dishes into a main meal of the day in place of a usual starchy side dish.

### 2.4. Resistant Starch Determination

Side dishes were heated in a 1100 W microwave for two minutes to mimic participant food preparation, except for the couscous and potato salad side dishes, which were to be served cold. Reheated side dishes were analyzed when dishes were warm; couscous and potato salad were analyzed chilled. Each sample was then ground evenly using an electric meat grinder (Aobosi, Guangzhou, China), on the “Fine” setting. RS was then immediately quantified by the Megazyme Resistant Starch Assay Kit (Megazyme, Bray, Ireland), according to the kit instructions. The Megazyme RS Assay Kit is able to analyze RS types 1, 2, and 3 and has a standard deviation of 3% and 3–5% between days according to the manufacturer. RS determination was done in triplicate for each study dish. Grams of RS were calculated by taking the percentage of RS wet weight and multiplying by the serving weight (in grams) of each dish.

### 2.5. Diet Quality

As detailed previously [25], participants completed five dietary recalls, one at baseline and one at the midpoint and end of each condition, using the Automated Self-Administered 24-h dietary recall (ASA-24) (National Cancer Institute, version 2016, Bethesda, MD, USA). These were scored using the Healthy Eating Index (HEI)-2015, a measure of adherence to the 2015–2020 Dietary Guidelines for Americans, with the code developed by the National Cancer Institute [26]. Exploratory correlations (Spearman) between baseline HEI-2015 total score and baseline fiber intake with baseline SCFA concentration were examined in SAS 9.4. *p*-values of <0.05 were used to determine statistical significance. Correlations with biochemical and vascular outcomes were not carried out as there were no significant changes in these outcomes [25].

### 2.6. Fecal Sample Collection

Study participants provided five fecal samples, one at baseline and one at the midpoint and end of each condition. Participants were instructed to collect the sample using the kit provided (stool hat, long handled spoon, medical gloves, clean vial); the specific collection protocol was explained, and written instructions were given. Participants were provided with coolers and ice packs and directed to freeze their sample immediately and bring it on ice within 24 h to the Clinical Research Center to be placed in a −80 °C freezer until analysis.

### 2.7. Short-Chain Fatty Acid Analysis

Fecal sample aliquots of approximately 0.25 g were thawed and diluted 1:5 with 10 mM H_2_SO_4_. Five 2 mm glass beads were added to each Eppendorf tube, and the samples were vortexed until fully homogenized. Tubes were then centrifuged at 5000 rpm for 15 min at room temperature. The supernatant was collected and centrifuged again at 13,000 rpm for another 15 min. The supernatant was collected and finally passed through a 0.45 µM filter. High-performance liquid chromatography (HPLC) was conducted using the Dionex ICS-5000+ DP equipped with Dionex VWD (Thermo Fisher, Waltham, MA, USA) for UV detection of SCFAs. A sample volume (from the preparations above) of 25 µL was injected into the system. The mobile phase was 5 mM H_2_SO_4_. Each run was 60 min of isocratic mobile phase flowing at 0.450 mL/min at 50 °C. Separation of SCFA was carried out with a guard column (Micro-Guard Cartridge Cation H+) and main column (Aminex HPX-87H) (Bio-Rad, Hercules, CA, USA). Peak sizes and fecal sample SCFA concentrations were calculated using the Chromeleon 7 analysis package (Thermo Fisher, Waltham, MA, USA), using standard calibration curves for each SCFA. A retention time of 20.01 min for acetate, 23.57 min for propionate, and 28.91 min for butyrate was used. Comparisons between conditions were conducted in SAS 9.4. Variables with non-normal distributions were log-transformed where appropriate. Mixed models were used to test the effect of condition, with subject as a random effect and condition as a fixed effect. The effect of order was included as a fixed effect, but it was removed from the final model as it was found not to significantly affect the outcome. Tukey–Kramer adjustments were used to account for multiple comparisons.

### 2.8. DNA Extractions and PCR Amplification

Fecal sample aliquots of approximately 0.25 g were thawed and placed in a bead beating tube from the QIAamp Powerfecal DNA Kit (Qiagen, Hilden, Germany). Next, 700 µL of the PowerBead solution was added to the bead beating tube before lysing in a BeadBeater (Biospec, Bartlesville, OK, USA) for 5 min at 3800 rpm. After lysing, the DNA extraction was completed using Qiagen PowerFecal kit according to kit instructions, until purified DNA was obtained. Extracted DNA of the V4 region of the 16S gene was amplified using forward primer 505F (5′-TCG TCG GCA GCG TCA GAT GTG TAT AAG AGA CAG GTG YCA GCM GCC GCG GA A-3′) and reverse primer 806R (5′-GTC TCG TGG GCT CGG AGA TCT GRA TAA GAG ACA GGG ACT ACN VGG GTW TCT AAT-3′). The PCR run conditions were as follows: 94 °C for 2 min, 30 cycles of 94 °C for 20 s, 56 °C for 30 s, 68 °C for 40 s, and finally, after cycle completion, 72 °C for 5 min.

PCR products were visualized on a 1% agarose gel stained with Midori Green DNA stain (Nippon Genetics Europe, Duren, Germany) to look for approximately 270 base pair bands. Once confirmed for desired base pair length, samples were sent to Pennsylvania State University’s Genomics Core Facility. Illumina adapters were added through a secondary PCR step. The PCR products were then normalized and purified before finally being loaded on an Illumina MiSeq V2 kit (Illumina, San Diego, CA, USA) for 250 × 250 nucleotide, paired-end sequencing.

### 2.9. Raw Sequence Processing

Detailed scripts used for processing can be found at: https://github.com/darrell25/DeMartino_Potato (accessed on 15 December 2021). Raw sequencing outputs were returned from the Genomics Core Facility as fastq files. Primer sequences were removed with the program cutadapt (v3.1) [27]. The trimmed sequencing reads were then processed in the program mothur, utilizing the MiSeq SOP method [28,29]. Forward and reverse reads were merged together, and reads of incorrect length or with ambiguous reads were screened out. Unique reads were then aligned to the Silva Database (version 132) [30]. Chimeric sequences were removed with U-CHIME [31]. The de novo OTUs were then generated using the opticlust algorithm at a cutoff of 0.03 or 97% similarity. Genus level taxonomy was assigned with the RDP classifier and version 18 of the RDP training set [32]. Blast+ (National Center for Biotechnology Information (NCBI)) [33] was used to assign species level taxonomy using the representative sequence of each OTU. Species names were only assigned if the BLAST hit had at least 97% identity, otherwise the genus level designation for the OTU was used. A phylogenetic tree for the de novo generated OTUs was produced using the program FastTree [34] with the representative FASTA sequence for each OTU. 

### 2.10. Diversity Analysis

Alpha diversity and beta diversity were analyzed at the genus level in R [35] utilizing the Phyloseq [36], Vegan [37], Picante [38], Microbiome [39], DivNet [40], and Breakaway [41] packages. All R code utilized in this analysis can be found in the GitHub repository referenced above. For all analyses, a Phyloseq object was first created consisting of a transposed version of the OTU table (shared file) generated by mothur, the taxonomy table generated by mothur updated with Blast+ species identifications and formatted for Phyloseq, the phylogenetic tree produced by FastTree, and a meta data file containing diet categories and information about the participants. 

For alpha diversity, Faith’s phylogenetic diversity, Shannon diversity and Inverse Simpson diversity were analyzed. For Faith’s phylogenetic diversity, the dataset was first rarefied to an even number of reads for each sample by random subsampling (4173, the minimum number of reads in any of the samples). The diversity was then calculated with the Picante package through the ‘pd’ function [38]. The resulting diversity measures were then compared between diet conditions with a linear mixed effect model with participant ID as a random effect using the lmer function of the lmerTest package [42]. For Shannon and Simpson diversity, the data were not rarefied and were instead determined via the DivNet package, which also generated confidence intervals for the individual measurements. These were then analyzed for significant differences between diet conditions, with the Breakaway package using participant ID as a random effect [40]. 

For beta diversity, weighted UniFrac, Bray–Curtis dissimilarity, and Aitchison distance analyses were performed. For weighted UniFrac analysis, the data were rarefied to an even number of reads (4173) for each sample by random subsampling. The analysis was performed using the ‘unifrac’ command in the Phyloseq package. Bray–Curtis distances were generated with the DivNet package. Aitchison distances were calculated by performing a centered-log-ratio transformation of the data with the Microbiome package, followed by calculation of Euclidian distance via the Vegan package. All beta diversity distance matrices were ordinated via principal coordinate analysis (Vegan ‘ordination’ function) and plotted to look for diet-based clustering. Diet-driven differences in beta diversity were statistically tested using the ‘adonis’ command in the Vegan package, which implements a PERMANOVA test [43]. 

### 2.11. Differential Abundance Analysis

Differential abundance analysis comparing the POT and REF dietary conditions was performed with 3 techniques with differing statistical approaches: LEfSe (non-parametric approach with relative abundances) [44], DeSEQ2 (Bayesian approach) [45], and ANCOM-II (log-ratio approach) [46]. In all cases, the taxa were first filtered for abundance (minimum 0.001% of total reads) and prevalence (present in at least 5% of samples). All *p*-values were corrected with the Benjamini–Hochberg method [47]. 

LEfSe was implemented through the Galaxy web application from the Huttenhower lab [48]. First, a Kruskal–Wallis test determined significantly abundant taxa at *p* < 0.05. Then, the effect size for each taxon was determined through a linear discriminant analysis (LDA) score. Taxa were considered differential at *p* < 0.05 and LDA score above 2.0. DeSEQ2 analysis was utilized through R using the DeSEQ2 package. ANCOM-II analysis [49] was performed in R using the implementation of Huang Lin [50]. Default parameters were used except that the zero_cut parameter (1—prevalence fraction) was adjusted to 0.95 to match the analysis performed with the other methods. Taxa were considered differentially abundant if they passed the 0.7 threshold of the method as it does not generate *p*-values.

## 3. Results

### 3.1. Participants

Fifty adults (68% female) aged 40 ± 13 years with an average BMI of 24.5 ± 3.6 kg/m^2^ completed this study. One participant dropped out of the study due to scheduling conflicts after baseline testing was performed. All participants who completed the study were included in analyses. One of the fecal samples did not generate any sequences during the sequencing run and had to be excluded from microbiota-based analyses, resulting in a total of 249 samples. 

### 3.2. Resistant Starch Analysis

RS was higher in the potato dishes compared to the refined grain dishes (POT: 1.31% wet basis (95% CI: 0.94, 1.71); REF: 0.73% wet basis (95% CI: 0.34, 1.14); *p* = 0.03). Potato study dishes ranged from 0.79% to 1.71% RS, while refined grain study dishes ranged from 0.1% to 1.9% RS (Table 1). Refined grain study dishes were more variable; white rice had the lowest RS (0.1%), while garlic bread had the highest (1.9%). Therefore, study participants were receiving on average an additional 1.74 g per day of RS when consuming the POT dishes vs. the REF dishes.

### 3.3. Short-Chain Fatty Acid Analysis

Butyrate, propionate, and acetate were not significantly different between conditions (Table 2), and there were no significant changes from baseline. Acetate was present in the greatest concentration compared to butyrate and propionate.

### 3.4. Alpha and Beta Diversity

**Figure 2:**
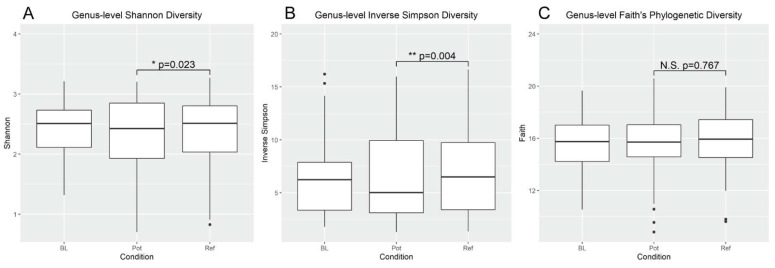
Alpha diversity analysis. The microbiotas of all participants were assessed for microbial diversity at baseline and during each diet condition. Shannon diversity, Inverse Simpson diversity, and Faith’s Phylogenetic Diversity were assessed for each. Statistical comparisons were made between the potato and refined grain conditions by a mixed effects model with participants used as a random effect. * indicates *p* < 0.05, ** indicates *p* < 0.01.

**Figure 3:**
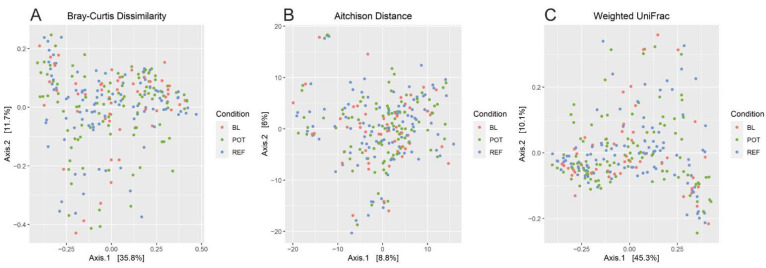
Beta diversity analysis. The microbiotas of all participants were assessed for between sample microbial diversity at baseline and during each diet condition. Bray–Curtis dissimilarity, Aitchison distance, and Weighted-UniFrac were used to assess the differences between the communities via principal coordinate analysis. Statistical comparison was made via PERMANOVA analysis. *p* < 0.05 was considered significant.

For alpha diversity, both Shannon diversity and inverse Simpson diversity were higher in the REF diet as compared to the POT diet, but the magnitude of the difference in both cases was small and very similar to baseline levels (Figure 2). There was no significant change observed in Faith’s phylogenetic diversity. For beta diversity as determined by Bray–Curtis, Aitchison and Weighted UniFrac, there were no clear separations by diet conditions in PCoA plots (Figure 3), and PERMANOVA analysis did not detect any significant differences by any of these measures. While the microbial communities did differ significantly by gender, age, and BMI, controlling for these variables did not reveal diet dependent effects at the community level. 

### 3.5. Correlations

There was a significant correlation between baseline HEI-2015 score and baseline acetate levels (rho = 0.36, *p* = 0.01), but not between baseline HEI-2015 and butyrate or propionate. No significant correlations were found between baseline dietary fiber intake and baseline SCFA. There were also no significant correlations found with SCFA and baseline fasting glucose or weight (Table 3).

### 3.6. Differential Abundance

**Figure 4:**
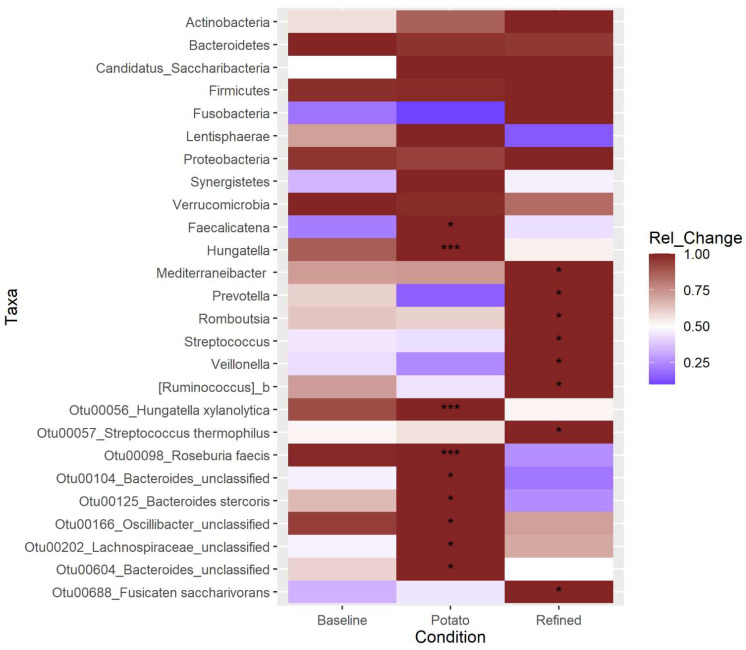
Differential abundance analysis. The heatmap is colored by the standardized change in the relative abundance of the taxon between the baseline and each of the diet conditions. Significant differences were calculated by three methods: LEfSe, DESeq2, and ANCOM-II. Significance is indicated by * for one method finding the taxon significantly different between that treatment and the control, *** for all three methods detecting a significant change. For LEfSe and DESeq2, adjusted *p*-values of 0.05 were used as the cutoff for significance, while for ANCOM-II, which does not generate *p*-values, the default cutoff threshold of 0.7 was used.

Differential abundance analysis using three methods (LEfSe, DESeq2, and ANCOM-II.) was conducted to test which taxa were enriched under each diet condition. Given the contrasting approaches each of these methods uses to measure differential abundance, taxa identified by two or more of these methods are more likely to be truly different between the conditions. Using this approach found no enriched taxa at the phylum level, eight enriched genera, and nine enriched OTUs (Figure 4). Of these, two OTUs, identified as *Hungatella xylanolytica* and *Roseburia faecis* were found to be enriched in the POT condition vs. REF by all three methods. Additionally, the *Hungatella* genus was found to be differentially abundant by all three methods, while the *Roseburia* genus was not found to be significantly changed. The *Hungatella xylanolytica* OTU was the only prominent member of that genus, while there are several *Roseburia* OTUs among the top 100 most abundant. While a number of other genera and OTUs were identified to be significantly enriched in one of the conditions, in all of these cases, it was only a single method that identified the difference, and more skepticism should therefore be applied. 

## 4. Discussion

In this study, intake of one potato-based dish per day, representing a relatively small increase in RS, induced an increase in abundances of *R. faecis* and *H. xylanolytica*, with a slight decrease in alpha diversity in comparison to intake of a refined grain-based dish. The potato dishes provided, on average, an additional 1.74 g of RS per day, far lower than has been previously tested in studies providing supplemental doses but representing a feasible dietary intake level for free-living individuals. Note that this still represents a substantial proportion of the average RS intake in the US, estimated at <6 g/day [21]. Raw potatoes contain far higher levels of RS, but much of this is lost during the cooking process. However, as this study has shown, significant amounts of RS2 can survive in these food products, and RS3 can be formed in dishes such as potato salad. It is also possible that the utilization of frozen dishes in this study led to the formation of RS3 in all dishes. Intriguingly, this small addition of RS to the daily diet led to detectable changes in the gut microbiota; the cross-over design of this study reduced the confounding effects of microbiota individuality due to factors such as age, gender, BMI, and many others. 

Few studies have examined the impacts of low doses of RS on the gut microbiota or the dose dependence of RS effects on the gut microbiota. Studies have been conducted in mice [51] and rats [52] examining the dose-dependent impacts of high amylose maize starch, an RS2. In these studies, impacts were still measurable in the lowest doses, but at 10% [52] and 18% [51] of the diet, the low doses in these studies still represent far higher levels of RS consumption than would be feasible for humans. In a recent study, Deehan et al. [53] examined doses ranging from 10 to 50 g/day of several RS4 (chemically modified starch) varieties in human volunteers. While changes in SCFA and diversity at the 10 g/day doses followed the trends seen at higher doses, they were not statistically significant. However, in some cases, differential abundance analysis revealed taxa-specific changes at the 10 g/day dose that were confirmed and strengthened at higher doses. It should be noted that the RS4 starches were particularly resistant, only slowly digested even by gut RS degrading bacteria in most cases. Thus, similar effects from more digestible RS2 starches might be expected at lower doses. Baxter et al. [18] reported increased fecal butyrate levels detected at a 14 g/day dose level for potato starch, though did not report on microbiota changes at this dose level. Furthermore, isolated prebiotic potato starch has been shown to significantly increase levels of *Bifidobacterium* [54,55], one of the most commonly reported effects of potato starch consumption on the gut microbiota [56]. In unpublished results, the same group has seen *Bifidobacterium* increases in doses as low as 3.5 g/day.

In the current study, the potato condition resulted in a decrease in both Shannon and Inverse Simpson diversity (though not Faith’s phylogenetic diversity) compared to the refined grain condition, which was not different from baseline for any of the alpha diversity measures. It is interesting that this low level of RS addition to the diet produced a detectable decrease in diversity. While we cannot say for certain that the changes to the microbiota induced by the potato dishes is due to the RS increase, this finding aligns with past RS supplementation studies, including with potato starch, though with much higher levels of RS [56]. It is likely that the relatively specialized nature of the community that responds to RS accounts for this diversity decrease, which may have detrimental impacts that somewhat offset the benefits of RS. Low levels of alpha diversity are associated with greater risk of low-grade chronic inflammation, obesity, and insulin resistance than higher levels of alpha diversity [57,58]. This may suggest that even low levels of RS supplementation would benefit from complementary dietary changes to maintain diversity, though it is possible that a decrease in diversity resulting from an increase in beneficial organisms is less detrimental. 

Diet quality in the TwinsUK cohort was evaluated using three indices of diet quality, including the Healthy Eating Index, and researchers showed that HEI was the best diet quality predictor of diversity in gut microbiota [59]. That study found that higher Mediterranean Diet scores and HEI scores were positively associated with greater levels of Shannon diversity. A study of 84 pregnant women with overweight and obesity also found that greater diet quality was associated with increased microbial diversity using the Shannon Index, and the bacterial species *Faecalibacterium prausnitzii* was also present in greater abundance in the group with higher diet quality [60]. We did not observe a significant correlation between baseline diet quality, as measured by the HEI-2015, and alpha diversity, likely due to the small sample size and the relatively healthy population being sampled. We did see a positive correlation between HEI-2015 and acetate levels in the gut, which as the most abundant of the SCFA may be a general proxy for overall fiber fermentation.

While the RS content of the participants’ background diets was not directly measured, minimal dietary changes were observed based on the 24 h recall data [25]. It is also important to note that throughout the study, participants were instructed to only consume the potatoes provided and no other potato-based products, making it easier to detect potato-induced changes when comparing the potato and refined grain conditions. Furthermore, past studies have generally found that microbiota changes induced by RS are unique to the source of RS [18,23,53], suggesting that background non-potato RS in the diet would have minimal impact on our ability to detect potato RS specific changes. It was expected that the most prominent signal from the gut microbiota in response to the addition of RS to the diet from the potato dishes would be from the known RS degrading bacteria in the human gut, *Bifidobacterium adolescentis* and *Ruminococcus bromii*. Past studies with potato starch, albeit at higher doses, have consistently noted increased levels of *B. adolescentis* in particular [56]. Neither of these bacteria were found to be significantly changed in this study, though they were the 9th (*B. adolescentis*) and 30th (*R. bromii*) most abundant OTUs overall, perhaps due to natural background fluctuations in these populations that masked their response to the RS. Two OTUs were found to increase in response to the potato diet, *Hungatella xylanolytica* and *Roseburia faecis*. Both *H. xylanolytica* (formerly *Bacteroides xylanolyticus*) and *R. faecis* are known starch degrading organisms [61,62]; however, while there are no reports of testing their growth on RS, neither of their genomes seem to contain the diverse set of extracellular starch digesting enzymes found in *B. adolescentis* or *R. bromii* for RS digestion [63,64,65]. Instead, these organisms are likely to be secondary participants in RS degradation, depending on *B. adolescentis* and *R. bromii* to initiate the process. It is likely that the complement of enzymes for starch digestion in *R. faecis* is similar to that found in the closely related and well characterized *Eubacterium rectale*. Despite having a complement of extracellular starch degrading enzymes and maltooligosaccharide transporters [66,67,68], *E. rectale* grows poorly on RS, and its enzymes are particularly ill suited to digesting intact potato starch. It is therefore likely that *R. faecis* is similarly dependent on a true RS degrading bacterium to initiate digestion, though like *E. rectale* (and unlike the RS degraders), it is a butyrate-producing bacterium that can contribute to some of the beneficial health effects seen with RS. The current study did not produce an increase in fecal butyrate levels; however, it is possible that butyrate production was slightly increased and compensated for by increased intestinal absorption.

In a companion study, 11 of the fecal samples from this study were used as the inoculum for in vitro fermentation experiments to test the abilities of these microbiotas to produce butyrate from a panel of resistant and non-resistant starches [20]. Looking at the results for the two individuals for which both species were detected in both the in vivo and in vitro experiments is potentially informative (Figure S1). For one of the subjects (Figure S1A,C), the potato side dish increased both species relative to the refined grain side dish, with similar levels of increase for potato starch granules during in vitro fermentation. However, both species increased more dramatically in cultures with retrograded potato starch. The same is true for *R. faecis* in the other subject (Figure S1B,D), though the trends are reversed for *H. xylanolytica* for this individual. This suggests that the presence of retrograded potato starch may be an important factor driving the changes seen. 

Intake of one potato-based side dish per day, containing 2.0–2.9 g RS compared to an isocaloric amount of refined grains in healthy adults, resulted in shifts in the gut microbiota. Abundance differences were found following both conditions for butyrate-producing and carbohydrate-degrading taxa,. however, no significant differences were observed in known RS degraders or fecal SCFA. The results of this study suggest that substituting one potato side dish for one refined grain side dish can influence the gut microbiota; however, the functional implications of these changes remain unclear. In conclusion, daily intake of one portion-controlled potato dish prepared in a healthy way increases RS intake and had measurable impact on the gut microbiota. Further research is required to understand the longer-term impacts of small increases in dietary RS on the gut microbiota.

## Figures and Tables

**Table 1:** Resistant starch content of the study dishes.

Potato Dishes	RS (% Wet Basis ± SE)	RS (Grams)	Refined Grain Dishes	RS (% Wet Basis ± SE)	RS (Grams)
Scalloped Potatoes	1.2 ± 0.05	2.7 g	Garlic Bread	1.9 ± 0.02	1.2 g
Smashed Potatoes	1.5 ± 0.07	2.9 g	Couscous Salad	0.6 ± 0.06	1.0 g
Roasted Paprika Potatoes	1.4 ± 0.03	2.1 g	Spanish Rice	0.1 ± 0.01	0.2 g
Herb Roasted Potatoes	1.7 ± 0.08	2.6 g	Red Pepper Rice	0.4 ± 0.04	0.7 g
Lemon Parsley Potatoes	1.7 ± 0.10	2.7 g	Naan	0.7 ± 0.61	0.6 g
Potato Salad	0.79 ± 0.04	2.1 g	Parmesan Orzo	0.7 ± 0.02	0.8 g
Potato and Spinach Casserole	1.0 ± 0.03	2.0 g	Mac’ n Cheese	0.7 ± 0.01	0.1 g
* Mean	1.33 ± 0.18	2.4 g	Mean	0.73 ± 0.18	0.66 g

Analyses were completed in triplicate, mean and SE presented here. RS analysis completed using Megazyme Resistant Starch Assay Kit (Megazyme, Bray, Ireland). * Means are significantly different, *p* = 0.03.

**Table 2:** Short-chain fatty acids, endpoint-to-endpoint means, and changes from baseline.

Short-Chain Fatty Acid	Baseline	Potato		Refined Grain		Change between Conditions	*p* *
		Endpoint	Change from Baseline	Endpoint	Change from Baseline		
Butyrate (mM)	13.6 (11.8, 15.3)	12.5 (10.7, 14.2)	−1.1 (−3.6, 1.4)	12.5 (10.8, 14.3)	−1.0 (−3.5, 1.5)	0.1 (−2.4, 2.6)	0.943
Propionate (mM)	12.1 (10.6, 13.6)	11.5 (9.9, 13.0)	−0.6 (−2.7, 1.4)	12.3 (10.8, 13.9)	0.2 (−1.8, 2.3)	0.9 (−1.1, 2.9)	0.704
Acetate (mM)	68.6 (62.1, 75.2)	68.3 (61.8, 74.7)	−0.4 (−10.7, 10.0)	72.6 (66.1, 79.1)	3.9 (−6.3, 14.3)	4.3 (−5.9, 14.6)	0.551

Data presented as LS means (95% CI). * Between condition differences.

**Table 3:** Baseline correlations between dietary intake, fasting glucose, weight, and SCFA and alpha diversity.

Correlations	Butyrate	Propionate	Acetate	Alpha Diversity (Shannon)
HEI-2015 ^a^	−0.13	0.15	0.36 *	−0.05
Fiber ^a^	−0.24	−0.16	−0.18	−0.07
FBG ^a^	−0.21	0.1	0.01	0.07
Weight ^a^	0.07	0.07	−0.02	0.15

^a^ values of the parameters used in this analysis come from [25]. Data presented as rho (Spearman correlation). * Significant correlation (*p* = 0.01).

## Data Availability

All microbiota sequence data were deposited into the NCBI sequence read archive under BioProject PRJNA780023. All code used in this manuscript can be found at: https://github.com/darrell25/DeMartino_Potato (accessed on 15 December 2021).

## Supplementary Materials

The following supporting information can be downloaded at: https://www.mdpi.com/article/10.3390/nu14030721/s1, Figure S1: Comparison between in vitro and in vivo studies.
