# Supplementary material for: Additional Resistant Starch from One Potato Side Dish per Day Alters the Gut Microbiota but Not Fecal Short-Chain Fatty Acid Concentrations
Source: Nutrients. 2022 Feb 8;14(3):721. doi: 10.3390/nu14030721 (PMC8840755; doi:10.3390/nu14030721)
Supplement: Supplementary file 1 [file nutrients-14-00721-s001.zip › nutrients-1541617-supplementary.pdf]

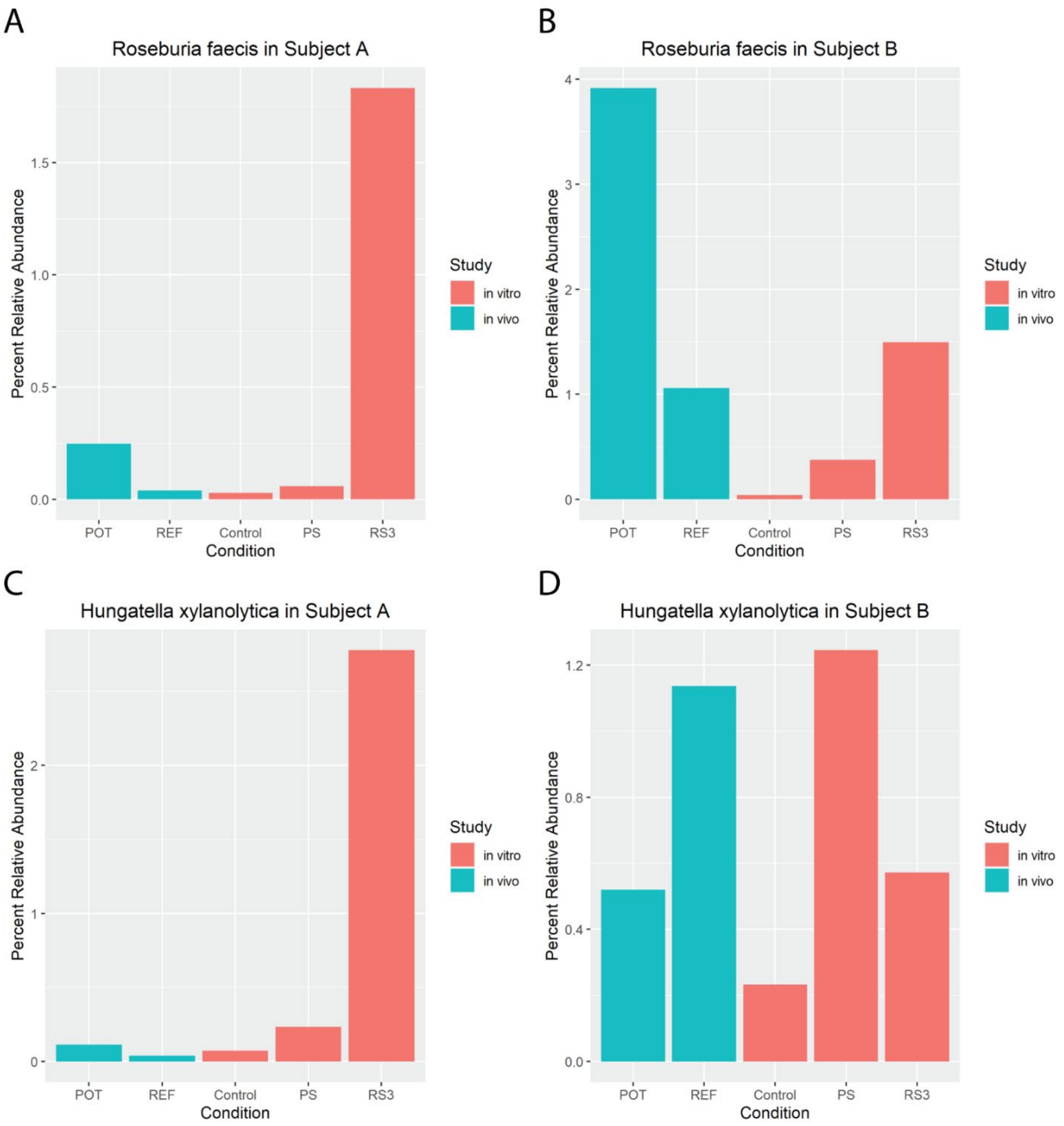

Figure S1. Comparison between in vitro and in vivo studies. The two OTUs (*Roseburia faecis* and *Hungatella xylanolytica*) that were found to be differentially abundant between the potato and re-fined grain side dish conditions were compared for two individuals for which in vitro fermentation studies had also been performed. The blue bars indicate the percent relative abundance for the in vivo study and the red bars for the in vitro study. A) is the amount of *R. faecis* found for Subject A, B) is the amount of *R. faecis* found in Subject B, C) is the amount of *H. xylanolytica* found for Subject A and D) is the amount of *H. xylanolytica* found in subject B. POT and REF are in vivo potato and refined conditions, respectively. Control, PS and RS3 are the in vitro control, granular potato starch and retrograded potato starch, respectively.
